# Comorbidity-stratified estimates of 30-day mortality risk by age for unvaccinated men and women with COVID-19: a population-based cohort study

**DOI:** 10.1186/s12889-023-15386-4

**Published:** 2023-03-13

**Authors:** Husam Abdel-Qadir, Peter C. Austin, Atul Sivaswamy, Anna Chu, Harindra C. Wijeysundera, Douglas S. Lee

**Affiliations:** 1 Women’s College Hospital, Toronto, ON Canada; 2 University Health Network, Toronto, ON Canada; 3 ICES (formerly known as the Institute for Clinical Evaluative Sciences), Toronto, ON Canada; 4 Institute of Health Policy, Management, and Evaluation, University of Toronto, Toronto, ON Canada; 5 Department of Medicine, University of Toronto, Toronto, ON Canada; 6 Sunnybrook Health Sciences Centre, Toronto, ON Canada; 7 Cardiovascular Research Program, Program Lead, ICES, 2075 Bayview Avenue, Room G-106, Toronto, ON M4N 3M5 Canada

**Keywords:** COVID-19, Vaccination, Chronic conditions, Frailty, Mortality

## Abstract

**Background:**

The mortality risk following COVID-19 diagnosis in men and women with common comorbidities at different ages has been difficult to communicate to the general public. The purpose of this study was to determine the age at which unvaccinated men and women with common comorbidities have a mortality risk which exceeds that of 75- and 65-year-old individuals in the general population (Phases 1b/1c thresholds of the Centre for Disease Control Vaccine Rollout Recommendations) following COVID-19 infection during the first wave.

**Methods:**

We conducted a population-based retrospective cohort study using linked administrative datasets in Ontario, Canada. We identified all community-dwelling adults diagnosed with COVID-19 between January 1 and October 31st, 2020. Exposures of interest were age (modelled using restricted cubic splines) and the following conditions: major cardiovascular disease (recent myocardial infarction or lifetime history of heart failure); 2) diabetes; 3) hypertension; 4) recent cancer; 5) chronic obstructive pulmonary disease; 6) Stages 4/5 chronic kidney disease (CKD); 7) frailty. Logistic regression in the full cohort was used to estimate the risk of 30-day mortality for 75- and 65-year-old individuals. Analyses were repeated after stratifying by sex and medical condition to determine the age at which 30-day morality risk in strata exceed that of the general population at ages 65 and 75 years.

**Results:**

We studied 52,429 individuals (median age 42 years; 52.5% women) of whom 417 (0.8%) died within 30 days. The 30-day mortality risk increased with age, male sex, and comorbidities. The 65- and 75-year-old mortality risks in the general population were exceeded at the youngest age by people with CKD, cancer, and frailty. Conversely, women aged < 65 years who had diabetes or hypertension did not have higher mortality than 65-year-olds in the general population. Most people with medical conditions (except for Stage 4–5 CKD) aged < 45 years had lower predicted mortality than the general population at age 65 years.

**Conclusion:**

The mortality risk in COVID-19 increases with age and comorbidity but the prognostic implications varied by sex and condition. These observations can support communication efforts and inform vaccine rollout in jurisdictions with limited vaccine supplies.

**Supplementary Information:**

The online version contains supplementary material available at 10.1186/s12889-023-15386-4.

## Background

It has been well-established that older age, male sex, and the presence of comorbidities are associated with higher mortality risks following diagnosis with the coronavirus disease of 2019 (COVID-19) [1–12]. Given COVID-19 incidence and mortality, and the precarious supply of COVID-19 vaccines globally [13–18], prioritization schemes will continue to be needed to triage vaccine delivery in early stages to people at the highest risk of death. When COVID-19 vaccines were first introduced in the United States, the Centre for Disease Control (CDC) Vaccine Rollout Recommendations [19] placed people aged 75 years and older in the Phase 1b stage, while Phase 1c extended eligibility to people aged 65–74 years and younger individuals with “underlying medical conditions which increase the risk of serious, life-threatening complications from COVID-19”.

However, this CDC approach treats “adults of any age” as being at increased risk of severe illness [20]. Thus, it does not account for differential risk between conditions, sex, or the multiplicative impact of older age on adverse outcomes among people with underlying medical conditions [21–26]. Other investigators have developed sophisticated risk prediction algorithms for mortality COVID-19 diagnosis [9, 27–32], but these do not lend themselves to simple implementation on a large scale by jurisdictions for vaccine prioritization. Furthermore, vaccine hesitancy remains an important stumbling block for vaccination in jurisdictions with adequate vaccine supplies. Unfortunately, the sociodemographic risk factors for chronic disease in the young overlap substantially with predictors of vaccine hesitancy [33–40]. Communication of risk for younger individuals can be hindered by lower absolute event rates, while relative risks can be harder to appreciate [41–45]. This has galvanized the development of alternate approaches for communication of risk for preventative intervention in younger patients [46–49].

Given these shortcomings, it would be desirable to provide relatively simple means to communicate how the risk of dying after being diagnosed with COVID-19 varies by age and sex for unvaccinated people living with comorbidity. Accordingly, we conducted a population-based cohort study of community-dwelling adults who developed COVID-19 before the availability of vaccines to quantify the incremental risk for death associated with underlying medical conditions as a function of age and sex. We specifically focused on cardiovascular disease, diabetes, hypertension, cancer, chronic obstructive pulmonary disease (COPD), chronic kidney disease (CKD) and frailty. We hypothesized that chronic medical conditions could elevate the risk of some, but not all, younger individuals to equal that of individuals aged > 65 years.

## Methods

### Study design and population

Residents of Ontario (Canada’s most populous province) receive universal coverage for essential physician services and hospital-based care through the Ontario Health Insurance Plan (OHIP). This facilitates the conduct of population-based cohort studies using administrative health datasets that are linked using unique encoded identifiers and are analyzed at ICES (formerly Institute for Clinical Evaluative Sciences). Multiple algorithms have been validated to ascertain medical diagnoses using these administrative databases [50]. ICES is an independent, non-profit research institute funded by an annual grant from the Ontario Ministry of Health (MOH) and the Ministry of Long-Term Care (MLTC), and a prescribed entity under Ontario’s Personal Health Information Protection Act (PHIPA), Sect. 45 of PHIPA. As such, the use of the data in this project is authorized under Sect. 45, approved by ICES’ Privacy and Legal Office, exempt from Research Ethics Board review, and does not require patient consent. All methods were carried out in accordance with locally relevant guidelines and regulations.

The Ontario Laboratories Information System (OLIS) was used to identify individuals aged ≥ 18 years with a positive reverse-transcription SARS-CoV-2 polymerase chain reaction (RT-PCR) test in Ontario between January 1 and October 31st, 2020, prior to the availability of vaccines. For people with more than one positive test, we retained the first positive test. The index date was that of collection of the qualifying SARS-CoV-2 swab. We excluded people with missing/invalid key data (age, sex, OHIP number), non-Ontario residents, OHIP coverage < 1 year before the SARS-CoV-2 test (for ascertainment of medical history), or an index positive SARS-CoV-2 test that was collected on a date when the individual was hospitalized (to limit our study to outpatients). We also excluded 5740 long-term care (LTC) residents (minimum age 20 years; maximum age 107 years) since they are already prioritized in the highest risk category globally (e.g., Phase 1a of the CDC framework). The remaining patients constituted our cohort of community-dwelling outpatients with COVID-19.

Our primary exposure was age. We also studied underlying medical conditions that have been shown to increase mortality risk in COVID-19 [1–12], affect a substantial proportion of the population, and are objective enough to be implemented by governments in vaccine prioritization policies: (1) major cardiovascular disease, defined as a recent (in past 5 years) myocardial infarction [51] or lifetime history of heart failure [52]; (2) diabetes [53]; (3) hypertension [54]; (4) cancer diagnosed within 5 years [55]; (5) COPD [56]; and (6) Stages 4/ 5 CKD (defined as dialysis-dependence or estimated glomerular filtration rate (eGFR) < 30ml/min/m2) [57]. For the analysis of CKD, we excluded individuals who were not dialysis-dependent and did not have creatinine measurements in the 2 years before contracting COVID-19. We also studied the Johns Hopkins ACG System binary frailty indicator [58] as a marker of global illness that can be applied widely across multiple health systems.

### Outcome

The primary outcome was death within 30 days of the positive SARS-Cov-2 test; this follow-up period after a positive PCR test is expected to capture most deaths directly attributable to COVID-19 [59, 60] while decreasing the likelihood of incorporating deaths due to other illnesses (which may be more likely for older patients with comorbidities).

### Statistical analysis

We fit univariable logistic regression models using Firth’s penalized likelihood approach to address potential bias in parameter estimates due to small sample and outcomes sizes in some age/condition strata [61, 62]. In these models, age was the only predictor, and modelled using a restricted cubic spline with knots at the 5th, 35th, 65th, and 95th percentiles, as suggested by Harrell to account for the non-linear relationship between age and our outcome [63] . Since separate curves are fit to each segment (i.e., range of ages), the model better reflects the relationship between age and death. The fitted model was used to estimate the risk of death within 30 days for 75- and 65-year-old individuals. These two probabilities were used as benchmarks against which to compare other subjects, since these are the age cut-offs used in Phases 1b and 1c respectively of the CDC’s COVID-19 Vaccine Rollout Recommendations. We then repeated the same logistic regression analyses, this time stratifying the cohort by sex and presence of the underlying medical conditions described above [62]. For each sex/comorbidity stratum, we determined the predicted risk of death within 30 days at all ages and identified the age at which individuals with the medical condition exceed the predicted benchmarks risk in the general population at age 65 years and 75 years. The age at which the benchmark risks were crossed were rounded up to the next integer for ease of presentation. All analyses were performed using SAS Enterprise Guide 7.1 (SAS Institute Inc., Cary, NC).

## Results

We studied 52,429 community-dwelling individuals who tested positive for SARS-CoV-2 between January 1 and October 31st, 2020 (Tables 1**and Supplemental Fig. 1**). Median age of our study population was 42 years [minimum, Q1, Q3, maximum 18, 29, 56, 104] years with 5,962 and 2,596 individuals 65- and 75 years and older, respectively; and 27,535 [52.5%] were women. A total of 1,185 individuals (2.3%) had major cardiovascular disease, 7,336 (14.0%) had been diagnosed with diabetes, 12,275 (23.4%) with hypertension, 966 (1.8%) with recent cancer, and 2,466 (4.7%) with COPD. Of 34,724 people (66.2% of cohort) whose CKD status could be ascertained, 350 (0.7%) were classified as having advanced CKD, of whom 146 (0.3%) were dialysis dependent. Using the Johns Hopkins indicator, 1,794 individuals (3.4%) were classified as frail.

**Table 1:** Baseline characteristics of study population by sex

Characteristic	Women	Men	Overall	Standardized difference	p-value
	N = 27,535	N = 24,894	N = 52,429		
Age in years, median (Q1, Q3)	43 (29, 56)	42 (29, 56)	42 (29, 56)	0.03	< 0.001
Major cardiovascular disease (recent myocardial infarction* or lifetime history of heart failure)	552 (2.0%)	633 (2.5%)	1,185 (2.3%)	0.04	< 0.001
Hypertension	6,288 (22.8%)	5,987 (24.0%)	12,275 (23.4%)	0.03	0.001
Diabetes	3,679 (13.4%)	3,657 (14.7%)	7,336 (14.0%)	0.04	< 0.001
Recent cancer*	563 (2.0%)	403 (1.6%)	966 (1.8%)	0.03	< 0.001
Chronic obstructive pulmonary disease (COPD)	1,236 (4.5%)	1,230 (4.9%)	2,466 (4.7%)	0.02	0.015
Stage 4/5 chronic kidney disease (CKD)†	167 (0.6%)	183 (0.7%)	350 (0.7%)	0.02	<0.001
Undetermined CKD status	8,070 (29.3%)	9,635 (38.7%)	17,705 (33.8%)	0.2	
Chronic dialysis	55 (0.2%)	91 (0.4%)	146 (0.3%)	0.03	< 0.001
Frailty‡	1,020 (3.7%)	774 (3.1%)	1,794 (3.4%)	0.03	< 0.001

* Hospitalization (for myocardial infarction) or diagnosis (for cancer) within the prior 5 years

† Defined as receiving chronic dialysis or eGFR < 30ml/min/m2* using the most recent serum creatinine result up to 2 years prior to positive SARS-CoV-2 test

‡ Defined using the Johns Hopkins frailty indicator from The Johns Hopkins ACG ® System Version 10.0

**Fig. 1:**
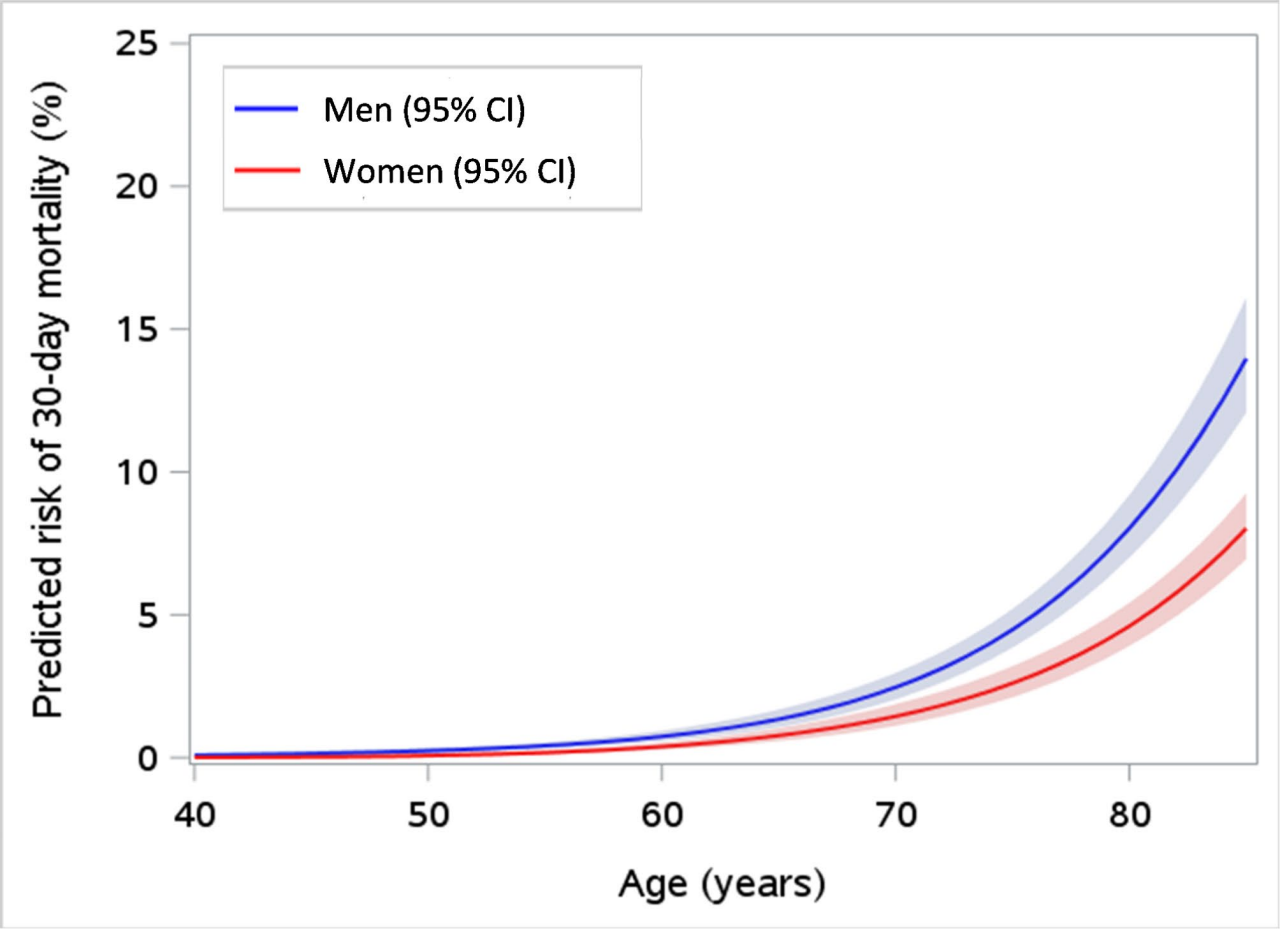
Predicted risk of 30-day death by age and sex. The shaded region indicates 95% confidence intervals. The blue curves depict estimated risk in men, while the red curves depict risk in women

Within 30 days following their positive SARS-CoV-2 test, 417 (0.8%) people died, with the greatest death rates for both men and women among those with hypertension (78,7% and 85.9%, respectively) or classified as frail (45.8% and 60.4%, respectively) (Table 2). The predicted risk of death in the general population was 1.1% at age 65 years and 3.4% at 75 years. The estimated 30-day mortality risk increased with age (1.7% among 65–74 year olds, and 11.5% among those 75 years and older), and was generally higher in men (Fig. 1). After stratifying by presence of underlying medical conditions, the estimated risk of death was generally higher in those with a comorbidity.

**Table 2:** Baseline characteristics of study population relative to sex and mortality status at 30 days following the qualifying positive SARS-CoV-2 test

Characteristic	Men			Women		
	No death within 30 days (n = 24,669)	Death within 30 days (n = 225)	Mortality rate (%) in men with condition	No death within 30 days (n = 27,343)	Death within 30 days (n = 192)	Mortality rate (%) in women with condition
Age in years, median (Q1, Q3)	41 (29, 56)	81 (68, 88)	-	43 (29, 56)	86 (79, 93)	-
Major cardiovascular disease (recent myocardial infarction* or lifetime history of heart failure)	569 (2.3%)	64 (28.4%)	10.1%	496 (1.8%)	56 (29.2%)	10.1%
Diabetes	3,558 (14.4%)	99 (44.0%)	2.7%	3,620 (13.2%)	59 (30.7%)	1.6%
Hypertension	5,810 (23.6%)	177 (78.7%)	3.0%	6,123 (22.4%)	165 (85.9%)	2.6%
Recent cancer*	380 (1.5%)	23 (10.2%)	5.7%	544 (2.0%)	19 (9.9%)	3.4%
Chronic obstructive pulmonary disease (COPD)	1,165 (4.7%)	65 (28.9%)	5.3%	1,178 (4.3%)	58 (30.2%)	4.7%
Stage 4/5 chronic kidney disease (CKD)†	156 (0.6%)	27 (12.0%)	14.8%	139 (0.5%)	28 (14.6%)	16.8%
Undetermined CKD status	9,629 (39.0%)	6 (2.7%)	0.1%	8,063 (29.5%)	7 (3.6%)	0.1%
Chronic dialysis	80 (0.3%)	11 (4.9%)	12.1%	48 (0.2%)	7 (3.6%)	12.7%
Frailty‡	671 (2.7%)	103 (45.8%)	13.3%	904 (3.3%)	116 (60.4%)	11.4%

* Hospitalization (for myocardial infarction) or diagnosis (for cancer) within the prior 5 years

† Defined as receiving chronic dialysis or eGFR < 30ml/min/m2* using the most recent serum creatinine result up to 2 years prior to positive SARS-CoV-2 test

‡ Defined using the Johns Hopkins frailty indicator from The Johns Hopkins ACG ® System Version 10.0

**Fig. 2:**
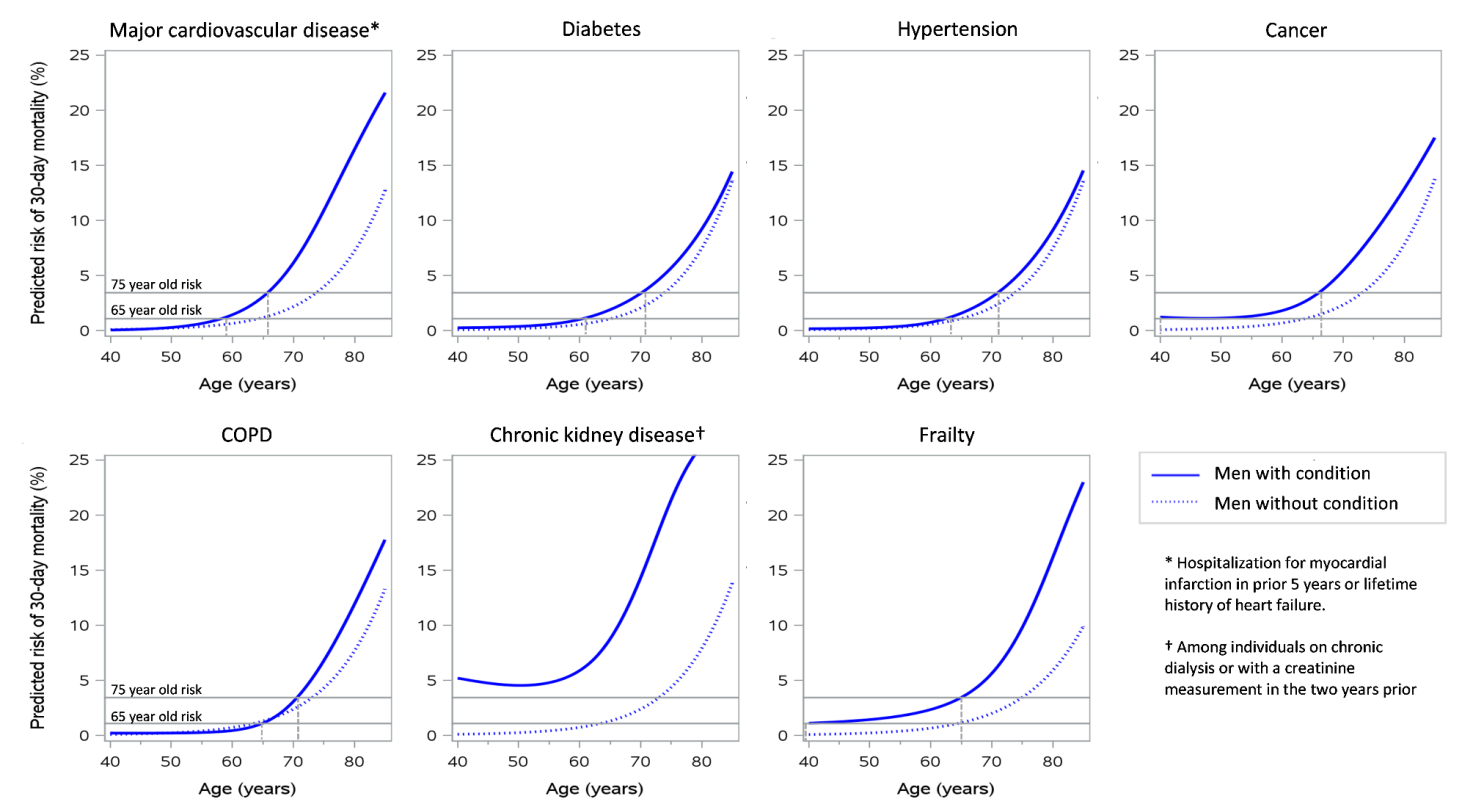
Predicted risk of 30-day mortality for men by presence of underlying medical conditions. Horizontal lines indicate the predicted risk of death in the general population at age 65 years and 75 years. Vertical lines highlight the age at which the risk for men with the medical conditions is equivalent to the general population aged 65- or 75- years

**Fig. 3:**
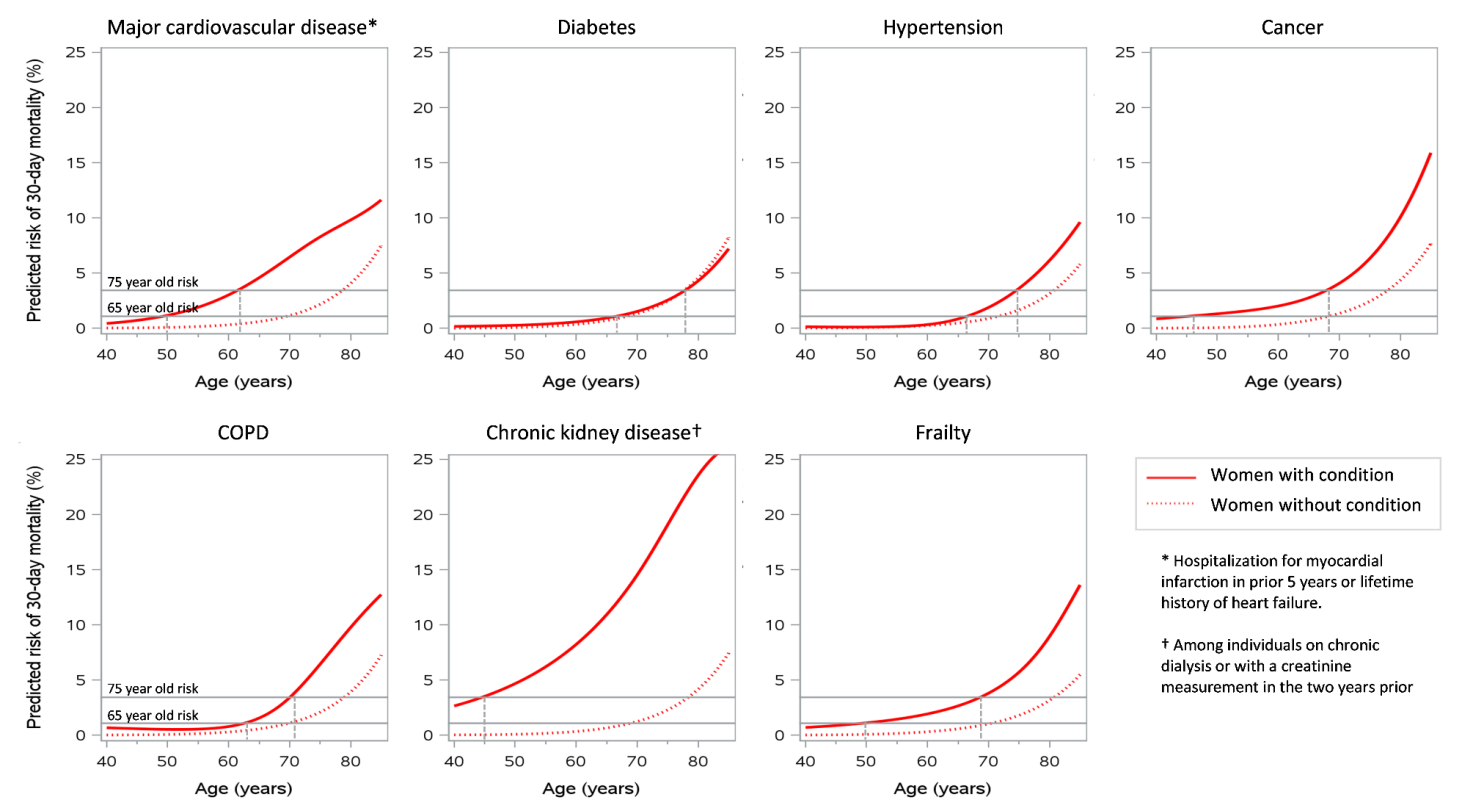
Predicted risk of 30-day mortality for women by presence of underlying medical conditions. Horizontal lines indicate the predicted risk of death in the general population at ages 65 years and 75 years. Vertical lines highlight the age at which the risk for women with the medical conditions is equivalent to the general population aged 65- or 75- years

Figures 2 and 3 illustrate the differences in risk of death at 30 days for men and women with and without underlying medical conditions. Overall, both men and women with a medical condition had higher 30-day death rates than those without, though a few exceptions were observed (i.e., among middle-aged men with versus without COPD and older women with versus without diabetes).

Table 3 summarizes the age (rounded up to the next integer) at which men and women with a particular comorbidity had the same predicted risk of death at 30-days as the general population aged 65 years and 75 years of age. The benchmark risks were exceeded at the earliest age by people with CKD, cancer, and frailty. For example, the risk of death at 30 days in men with CKD at age < 40 years and women with CKD at age 45 years equalled the risk of 75-year-olds from the general population. In contrast, the risk of death at 30 days for women aged < 65 years who had diabetes or hypertension was not higher than the 30-day mortality risk of 65-year-olds in the general population.

**Table 3:** Age at which the risk of death in men and women with an underlying medical condition exceeds the risk in the general population aged 65- and 75-years of age. The presented age has been rounded up to the next integer

		Age with equivalent risk to a 65-year-old in the general population	Age with equivalent risk to a 75-year-old in the general population
Major cardiovascular disease (recent myocardial infarction* or lifetime history of heart failure)	Men	59	66
	Women	50	62
**Diabetes**	Men	61	71
	Women	67	78
**Hypertension**	Men	63	71
	Women	67	75
Recent cancer*	Men	< 40‡	66
	Women	46	68
**Chronic obstructive pulmonary disease (COPD)**	Men	65	71
	Women	63	71
**Stage 4/5 chronic kidney disease (CKD)†**	Men	< 40‡	< 40‡
	Women	< 40‡	45
**Frailty, as defined by Johns Hopkins indicator**	Men	39	65
	Women	50	69

* Hospitalization (for myocardial infarction) or diagnosis (for cancer) within the prior 5 years

† Defined as receiving chronic dialysis or eGFR < 30ml/min/m2* using the most recent serum creatinine result up to 2 years prior to positive SARS-CoV-2 test

‡ Suppressed due to the small population size or number of events in the sex/condition stratum

## Discussion

In this population-based cohort study, we determined the estimated age at which a community-dwelling man or woman with underlying medical conditions will exceed the 30-day mortality risk of the typical person included in Phase 1b or 1c of the CDC recommendations based on age alone. While the risk of death after COVID-19 was higher in people with underlying medical conditions, the prognostic implications varied by sex and condition. The risk of 30-day death was generally higher in men than in women. The increase in risk incurred by the presence of CKD and recent cancer was higher than isolated diabetes or hypertension. Thus, it would be inappropriate to treat hypertension and diabetes as being equivalent to the other comorbidities studied when triaging vaccine rollout. The Johns Hopkins ACG System binary frailty indicator was a useful composite measure which can be applied by large health systems using administrative data for identification of individuals at higher risk for death with COVID-19 despite younger age.

Numerous publications have demonstrated that individuals with comorbidities are at higher risk for adverse outcomes following COVID-19 infection [1–12]. The comorbidities studied in our analysis have been among the most studied and most consistently linked to higher mortality with COVID-19 [1, 2, 6, 11]. In meta-analyses of studies prior to vaccine availability, reported risk estimates have ranged from 3.07 to 4.90 for CKD, 1.47 to 1.90 for cancer, and 2.25–3.05 for cardiovascular disease [1, 6, 11]. Male sex has also been consistently shown to increase the risk of adverse outcomes following COVID-19 infection [5, 8, 9], which may be related, in part, to the X-linked nature of the SARS-CoV-2 receptor [64]. The combination of multiple comorbidities increase risk even further, and this has been utilized to develop comprehensive models which predict the risk of death following COVID-19 infection with high accuracy [9, 27–32]. In a study of Veteran Affairs data in the United States, a model of nine risk factors including age, sex, diabetes, CKD and heart failure demonstrated a discriminative ability of 83.4% compared to 74.0% in a model using age alone, as in the CDC approach [65]. Furthermore, once population vaccination rates reach 50%, vaccine prioritization based on the model was estimated to result in 21.5% fewer deaths than prioritization on the CDC phased approach [65].

However, while these studies have provided valuable information about factors associated with increased COVID-19 mortality, we are not aware of studies reporting their age-equivalent mortality risk. Additionally, the inferences from these studies about the impact of comorbidity and sex on the risk of dying from COVID-19 at different ages are challenging to communicate to the public in a transparent and easily understood manner [41–43]. The communication gap is expected to be largest for demographic groups that are at higher risk for COVID-19 and more susceptible to misinformation [66–68]. The profound impact of some comorbidities on mortality is important to communicate to younger individuals with vaccine hesitancy, which remains an important issue among people with comorbidity, particularly those living in communities in which medical comorbidity is more likely to emerge at a younger age [33–40]. Provision of visual cues and expressing risk in terms of relative age (e.g., “heart age”) improves communication of cardiovascular risk for younger individuals and is more likely to promote behaviour change than traditional methods of communicating cardiovascular risk [42–49, 69]. We present our data in an analogous approach, which we believe can be helpful for vaccine-hesitant individuals with comorbidities that confer higher risk of adverse outcomes following COVID-19.

Importantly, we showed that regardless of underlying medical condition, the mortality risk rises substantially with age, meaning that age should continue to be a key factor in the triage process. The presence of most underlying medical conditions in individuals under the age of 45 years did not elevate their risk to that of the general population at age 65 years, with the notable exception of Stage 4–5 CKD, and among men, recent cancer and frailty. In other words, the protective effects of younger age persisted despite the presence of underlying medical conditions for most people aged < 45 years.

Several limitations to our study are noted. By design, we adopted an approach prioritizing parsimony and simplicity by focusing on the presence/ absence of comorbidity without accounting for the severity of disease. Although 12.8% of our study population had more than one of the conditions studied, we also did not study the combined impact of multiple comorbidities outside the frailty indicator for the same reasons. As a result, in our comparisons of risk among men and women with specific conditions versus without, it is possible that some differences may be attenuated because those without the condition have other conditions which also increase their risk. Another limitation was the small event counts for sex-specific strata of patients with cancer and CKD, which decreases precision of our estimates for these groups. Our results also do not apply to LTC residents, who should already be considered at highest risk regardless of age or comorbidities. Finally, since our study population was identified during the first eight months of the pandemic and prior to the appearance of variants of concern, such as Delta and Omicron, the absolute risk estimates by age may not be applicable to patients diagnosed with these newer variants; however, the higher mortality risks associated with CKD, cancer and frailty compared with hypertension and diabetes are likely applicable beyond the first wave though the relative risk associated with variant types differs.[70, 71] Additionally, the relative ages reported from our analyses remain valuable for communication of relative risks.

## Conclusion

The mortality risk in COVID-19 increases with age and comorbidity but the prognostic implications varied by sex and condition. The risk was generally higher for men, and the increase in risk associated with CKD, cancer, and frailty was higher than what was observed with hypertension and diabetes. We hope to improve communication by presenting the combined impact of patient age, sex, and comorbidity using visual scales and reporting the “equivalent age” at which individuals would have the same mortality risk as the average 65- and 75-year-old with COVID-19. We believe these observations can inform vaccine rollout and support communication efforts in jurisdictions with limited vaccine supplies.

## Electronic supplementary material

Below is the link to the electronic supplementary material.

Supplementary Material 1

## Data Availability

The dataset from this study is held securely in coded form at ICES. While legal data sharing agreements between ICES and data providers (e.g., healthcare organizations and government) prohibit ICES from making the dataset publicly available, access may be granted to those who meet pre-specified criteria for confidential access, available at www.ices.on.ca/DAS (or email Data Analytic Services at das@ices.on.ca). The full dataset creation plan and underlying analytic code are available from the corresponding author, Douglas S. Lee (Douglas.Lee@ices.on.ca) upon request, understanding that the computer programs may rely upon coding templates or macros that are unique to ICES and are therefore either inaccessible or may require modification.

## Abbreviations

CDC: Centre for Disease Control
CKD: Chronic kidney disease
COPD: Chronic obstructive pulmonary disease
eGFR: Estimated glomerular filtration rate
LTC: Long-term care
OHIP: Ontario Health Insurance Plan
OLIS: Ontario Laboratories Information System
RT-PCR: Reverse-transcription SARS-CoV-2 polymerase chain reaction
