# Supplementary material for: Comorbidity-stratified estimates of 30-day mortality risk by age for unvaccinated men and women with COVID-19: a population-based cohort study
Source: BMC Public Health. 2023 Mar 13;23:482. doi: 10.1186/s12889-023-15386-4 (PMC10010246; doi:10.1186/s12889-023-15386-4)
Supplement: Supplementary file 1 — Supplementary Material 1 [file 12889_2023_15386_MOESM1_ESM.docx]

**Supplemental Figure 1. Cohort creation flow diagram**

**
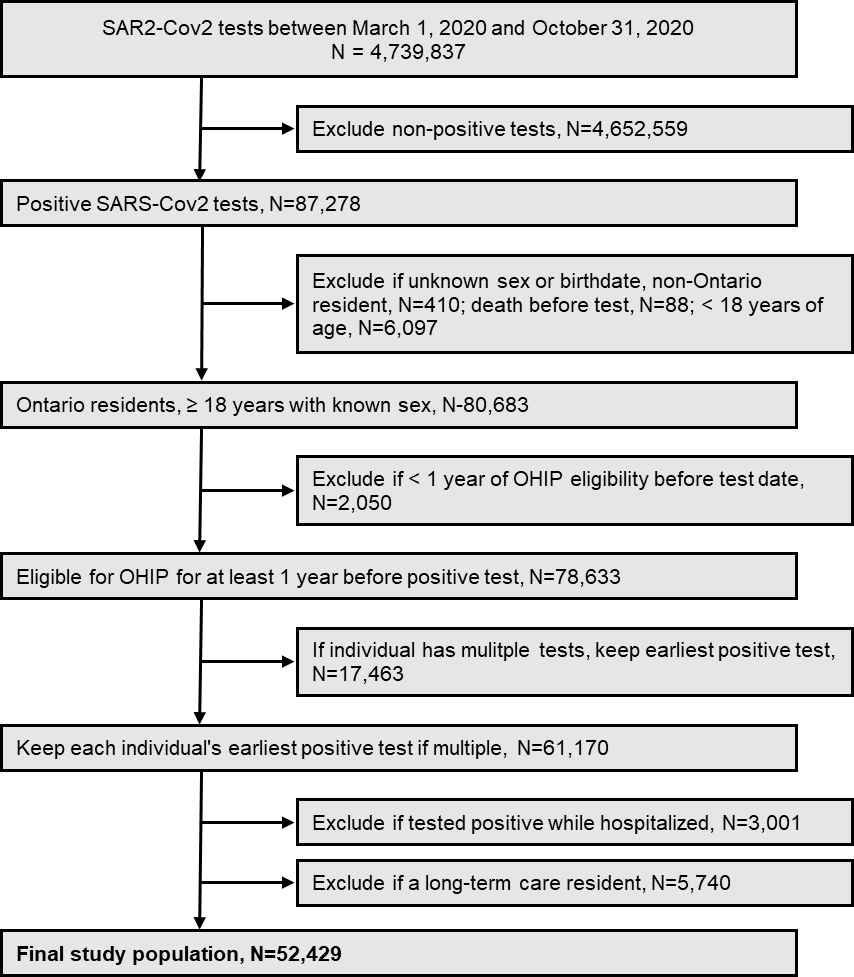
**

Abbreviations: OHIP, Ontario Health Insurance Plan.
